# Mucosal Gene Expression of Antimicrobial Peptides in Inflammatory Bowel Disease Before and After First Infliximab Treatment

**DOI:** 10.1371/journal.pone.0007984

**Published:** 2009-11-24

**Authors:** Ingrid Arijs, Gert De Hertogh, Katleen Lemaire, Roel Quintens, Leentje Van Lommel, Kristel Van Steen, Peter Leemans, Isabelle Cleynen, Gert Van Assche, Séverine Vermeire, Karel Geboes, Frans Schuit, Paul Rutgeerts

**Affiliations:** 1 Department of Gastroenterology, University Hospital Gasthuisberg, Leuven, Belgium; 2 Gene Expression Unit, Department of Molecular Cell Biology, Katholieke Universiteit Leuven, Leuven, Belgium; 3 Department of Morphology and Molecular Pathology, University Hospital Gasthuisberg, Leuven, Belgium; 4 Department of Electrical Engineering and Computer Science (Montefiore Institute), University of Liege, Liege, Belgium; 5 Leuven Food Science and Nutrition Research Centre (LFoRCe), University Hospital Gasthuisberg, Leuven, Belgium; HelmholtzZentrum München, Germany

## Abstract

**Background:**

Antimicrobial peptides (AMPs) protect the host intestinal mucosa against microorganisms. Abnormal expression of defensins was shown in inflammatory bowel disease (IBD), but it is not clear whether this is a primary defect. We investigated the impact of anti-inflammatory therapy with infliximab on the mucosal gene expression of AMPs in IBD.

**Methodology/Principal Findings:**

Mucosal gene expression of 81 AMPs was assessed in 61 IBD patients before and 4–6 weeks after their first infliximab infusion and in 12 control patients, using Affymetrix arrays. Quantitative real-time reverse-transcription PCR and immunohistochemistry were used to confirm microarray data. The dysregulation of many AMPs in colonic IBD in comparison with control colons was widely restored by infliximab therapy, and only *DEFB1* expression remained significantly decreased after therapy in the colonic mucosa of IBD responders to infliximab. In ileal Crohn's disease (CD), expression of two neuropeptides with antimicrobial activity, *PYY* and *CHGB*, was significantly decreased before therapy compared to control ileums, and ileal *PYY* expression remained significantly decreased after therapy in CD responders. Expression of the downregulated AMPs before and after treatment (*DEFB1* and *PYY*) correlated with villin 1 expression, a gut epithelial cell marker, indicating that the decrease is a consequence of epithelial damage.

**Conclusions/Significance:**

Our study shows that the dysregulation of AMPs in IBD mucosa is the consequence of inflammation, but may be responsible for perpetuation of inflammation due to ineffective clearance of microorganisms.

## Introduction

Inflammatory bowel diseases (IBDs), Crohn's disease (CD) and ulcerative colitis (UC), are multifactorial diseases of unknown etiology, characterized by chronic relapsing inflammation of the gastro-intestinal tract. Immune, genetic and environmental factors are thought to contribute to IBD [1].

Many findings suggest that the intestinal flora plays an important role in the pathogenesis of IBD. First, several knockout animal models of IBD in a germ-free environment fail to develop intestinal inflammation [2]–[6]. Second, recurrence of CD in the neoterminal ileum after ileal resection with ileocolonic anatomosis has been shown to be dependent on faecal stream [7]. Third, luminal contents trigger inflammation [8], and T-cell responses in CD patients are directed against the autologous bacterial flora [9]. Fourth, in CD mucosa adherent *Escherichia coli* has been found [10]. Finally, antibiotics and probiotics do often ameliorate the symptoms in IBD [11].

Together these points support the microbial contribution to IBD, indicating that a basic antimicrobial mucosal barrier defect could be responsible for susceptibility to this disease. The gastro-intestinal tract is constantly exposed to a wide range of microorganisms. In order to maintain the mucosal barrier integrity against these luminal microorganisms, the intestinal epithelial cells produce a variety of antimicrobial peptides (AMPs), like defensins, lysozyme and cathelicidins. AMPs contribute to innate immunity and can be considered as natural peptide antibiotics. Several recent studies indicate that an abnormal expression of AMPs may exist in IBD [12]. Patients with Crohn's ileitis (CDi) show a reduced antibacterial activity in their intestinal mucosal extracts and display a decreased expression of the Paneth cell alpha-defensins (*DEFA5* and *DEFA6*) [13]. In the study by Wehkamp et al. [14], this decrease was found independent of the degree of inflammation and associated with *NOD2* mutations. However, another study found no correlation between the decreased alpha-defensins expression and the *NOD2* status but linked the decrease to inflammation [15]. A study by Noble et al. [16] showed a regional variation of *DEFA5* and *DEFA6* gene expression in non-inflamed intestinal biopsies of normal subjects and UC patients, with high expression in the terminal ileum and expression decreasing as the biopsy location became more distal in the colon. They further found a marked upregulation of *DEFA5* and *DEFA6* expression in inflamed UC colon. As compared to UC, Crohn's colitis (CDc) is characterized by a decreased antimicrobial activity in cationic protein extracts from colonic biopsies and an attenuated induction of beta-defensins (*DEFB4/HBD-2*, *DEFB103*), cathelicidin *LL37*, and antimicrobial antiproteases elafin and *SLPI* [17]–[21]. The defective mRNA induction of beta-defensins in CDc may be partly due to low *DEFB4* gene copy number [22]. However, no association with *DEFB4* gene copy number was found on DEFB4 protein level [23].The *DEFB1* expression was found to be decreased in both active UC and CD [18].

It can be argued, however, that the abnormal AMP status of IBD patients is the consequence of an altered interaction between barrier and microflora interaction rather than a causative factor for disturbed microbial clearance. The hypothesis for the present study was that disturbed AMP expression in IBD is a pathogenic factor. When true, the prediction is that after pharmacological suppression of inflammation the underlying defective expression of AMPs would be unmasked. However, if the alternative hypothesis is true, namely that abnormalities in AMP production are a secondary phenomenon, it can be predicted that after disappearance of inflammation the AMP expression in IBD normalizes. To distinguish between these two hypotheses, we investigated the intestinal mucosal gene expression of AMPs in active IBD patients and the impact of anti-inflammatory therapy with infliximab (Remicade; Centocor, Inc., Malvern, PA, USA), a chimeric antibody against tumor necrosis factor-alpha (TNF-alpha), on the mucosal gene expression of AMPs in IBD patients, using microarray technology.

## Methods

### Ethics statement

The study was carried out at the University Hospital of Gasthuisberg in Leuven (ClinicalTrials.gov number, NCT00639821). The ethics committee of the University Hospital approved the study and all individuals gave written informed consent.

### Antimicrobial peptides

The name AMPs is somewhat confusing and large number of very diverse proteins has been given this name in the literature. There are the “classic” AMPs that were discovered for their action to kill microorganisms (e.g. defensins and cathelicidins), and other proteins that were discovered for other biological functions but they were reported to exert antimicrobial activities (e.g. neuropeptides, chemokines and proteinase inhibitors) [24].

The PubMed database was searched using the keywords “antimicrobial peptide” and “antimicrobial protein”, and 81 peptides/proteins with reported antimicrobial activity were selected and analysed in this study (Table S1). We excluded the AMPs that were identified as chemokines [25] because of their key role in the inflammatory response in IBD.

### Patients and biopsy specimens

Sixty-one patients with active IBD (24 UC, 19 CDc and 18 CDi), refractory to corticosteroids and/or immunosuppression, and a control group of 12 individuals (6 colon and 6 ileum) who underwent endoscopy for screening for polyps were studied. The patients underwent endoscopy with biopsies from diseased bowel (colon for UC and CDc, and ileum for CDi) within a week prior to the first intravenous infusion of 5 mg infliximab per kg body weight. They underwent a second endoscopy with biopsies 4 weeks after the first infliximab infusion in case of a single infusion and at 6 weeks if they received a loading dose of infliximab at weeks 0, 2 and 6. The biopsies were taken at sites of active inflammation but at a distance of ulcerations. In the case of healing at control endoscopy, the biopsies were obtained in the areas where lesions were present before therapy. The endoscopist was not blinded to treatment. Half of the biopsies were immediately snap-frozen in liquid nitrogen and stored at −80°C until RNA isolation and/or immunohistochemistry, except for the biopsies from 1 CDc patient after infliximab treatment which were of poor technical quality. The residual biopsies were fixed in Carnoy's fixative for up to 5 hours and then dehydrated, cleared and paraffin-embedded for histologic examination. The features of chronic intestinal inflammation were scored in haematoxylin-eosin stained slides from the paraffin blocks of each patient using a previously reported scoring system for UC [26] and for CD [8]. The pathologists who scored the biopsies (KG and GDH) were blinded to treatment.

The response to infliximab was assessed 4 to 6 weeks after the first infliximab treatment. For UC and CDc, the response to infliximab was defined as a complete mucosal healing with a decrease of at least 3 points on the histological score for CDc [8] and as a decrease to a Mayo endoscopic subscore of 0 or 1 with a decrease to grade 0 or 1 on the histological score for UC [26], [27]. Patients who did not achieve this healing were considered non-responders although some of them presented endoscopic and/or histologic improvement. Of the 43 colonic IBD (IBDc) patients, we identified 20 responders (8 UC and 12 CDc) and 23 non-responders (16 UC and 7 CDc). If the same response criteria of CDc were used for CDi, only one patient showed complete endoscopic and histologic healing. Therefore, we had to use less strict response criteria for CDi. Patients with a clear improvement of the ulcerations and a decrease on the histological score [8] were considered responders. Of the 18 CDi patients, we identified 8 (partial) responders and 10 non-responders.

The baseline characteristics of the patients are summarized in table 1.

**Table 1:** Baseline characteristics of the UC, CDc and CDi patients.

Characteristics	UC (n = 24)	CDc (n = 19)	CDi (n = 18)
Male/Female (%)	14/10 (58.3/41.7)	11/8 (57.9/42.1)	9/9 (50/50)
Median (IQR) age at first IFX (years)	41.4 (31.9–50.9)	31.8 (23.7–47.5)	46.4 (34–55.3)
Median (IQR) weight at first IFX (kg)	72.5 (67–80.3)	68 (60.5–77.5)	63.5 (56.1–79.5)
Median (IQR) duration of disease prior to first IFX (years)	7.3 (2.7–17.1)	6.4 (3.1–20.9)	22.3 (11.1–28)
Extent of disease			
UC Left-sided colitis (%)	7 (29.2)	NA	NA
Pancolitis (%)	17 (70.8)	NA	NA
CD Ileocolon (%)	NA	5 (26.3)	9 (50)
Ileum (%)	NA	0 (0)	9 (50)
Colon (%)	NA	14 (73.7)	0 (0)
Median (IQR) C-reactive protein at first IFX (mg/dL)	4 (1.8–19.1)	10.2 (4.3–35)	7.4 (2.3–10.9)
Concomitant medication at first IFX (%)			
5-Aminosalicylates	18 (75)	8 (42.1)	5 (27.8)
Corticosteroids	7 (29.2)	4 (21.1)	2 (11.1)
Azathioprine/6-Mercaptopurine	15 (62.5)	14 (73.7)	7 (38.9)
Methotrexate	0 (0)	0 (0)	0 (0)
Corticosteroids+Immunosuppressants	3 (12.5)	2 (10.5)	1 (6)
Active smoking at first IFX (%)	2 (8.3)	6 (31.6)	6 (33.3)

IQR, interquartile range; IFX, infliximab; NA, not applicable.

### RNA isolation and oligonucleotide array hybridization

Total RNA was extracted from the biopsy specimens using the RNeasy Mini Kit (Qiagen, Benelux B.V.), according to the manufacturer's instructions. The integrity and quantity of total RNA were assessed with a 2100 Bioanalyzer (Agilent, Waldbronn, Germany) and Nanodrop ND-1000 spectrophotometer (Nanodrop technologies). As previously described [28], total RNA was analyzed with the Affymetrix Human Genome U133 Plus 2.0 Arrays (Affymetrix, Santa Clara, CA, USA), which comprised of 54675 probe sets covering the whole genome. The microarray data were deposited at Gene Expression Omnibus under the series accession number GSE16879, and the microarray data were handled in accordance with the MIAME (Minimum Information About a Microarray Experiment) guidelines.

### Microarray data analysis

A glossary of terms used in the analysis is provided in Appendix S1.

The Affymetrix raw data (.cel files) were analyzed using Bioconductor tools [29] in R (version 2.7.2, http://www.r-project.org/). The robust multichip average method [30] was performed on the Affymetrix raw data (.cel files) to obtain a log2 expression value for each probe set. Probe set annotations were obtained through the Affymetrix NetAffx website (http://www.affymetrix.com/analysis/index.affx) or the UCSC Genome Browser website (http://genome.ucsc.edu/) or the NCBI website (http://www.ncbi.nlm.nih.gov/). For comparative analysis, linear models for microarray data (LIMMA) [31] was performed for all the probe sets (54675 probe sets) present on the microarray to identify probe sets that are different between the groups, based on moderated *t*-statistics. To correct for multiple testing, the false discovery rate (FDR) was estimated from p-values derived from the moderated *t*-statistics using the method of Benjamini and Hochberg [32]. Probe sets with a>2-fold change (FC) and a FDR<0.05 were considered biologically significant. In this study, we focused on the microarray data of AMP genes. We selected the results from all performed comparative analyses for the probe sets encoding AMP genes, interleukin 8 (*IL8*) gene (probe set 202859_x_at) and villin 1 (*VIL1*) gene (probe set 205506_at) (Table S2).

### Quantitative real-time reverse-transcription PCR (qPCR)

To validate the microarray data, qPCR was performed for *DEFA5*, *DEFA6*, *DEFB4*, *DEFB1*, liver expressed antimicrobial peptide 2 (*LEAP2*) and peptide YY (*PYY*). Beta-actin was used as the endogenous reference gene. Total RNA from the same samples as for microarray analysis was used. cDNA was synthesized from 0.5 µg of total RNA using the RevertAid H Minus First Strand cDNA synthesis kit (Fermentas, St. Leon-Rot, Germany), following the manufacturer's protocol. Primers and dual-labeled probes were designed using OligoAnalyzer 3.0 software (http://biotools.idtdna.com/analyzer/) and synthesized by Sigma-Genosys (Haverhill, UK). The oligonucleotide sequences are available upon request. Multiplex real-time PCR was performed in a final reaction volume of 25 µl on a Rotor-Gene 3000 instrument (Corbett Research, Mortlake, Australia), using QuantiTect Multiplex PCR NoROX Kit (Qiagen, Venlo, NL), according to the manufacturer's instructions. Cycle threshold values were determined by Rotor-Gene 6.0.16 software. All samples were amplified in duplicate reactions. The relative expression of target mRNA levels were calculated as a ratio relative to the beta-actin reference mRNA [33]. Results were analyzed using the Mann-Whitney *U*-test for unpaired samples and Wilcoxon signed-rank test for paired samples using SPSS 16.0 software (SPSS, Chicago, IL). A p-value of <0.05 was considered significant.

### Immunohistochemistry

To determine the protein localization of lysozyme (LYZ), DEFA5 and DEFB1, immunohistochemical staining was performed on 5 µm-thick cryostat sections from fresh-frozen intestinal mucosal biopsies obtained during endoscopy from the IBD patients and the control individuals. All procedures were conducted at room temperature. Briefly, cryostat sections were air-dried overnight after they were cut, fixed in acetone for 10 min and rinsed in phosphate-buffered saline (PBS) for 5 min. Sections were pre-treated with Dual Endogenous Enzyme-Block Reagent (Dako Belgium nv/sa, Heverlee, Belgium) for 10 min, washed in PBS and incubated for 30 min with the primary antibody (Ab) against LYZ (rabbit polyclonal anti-human lysozyme Ab, EC 3.2.1.17, code A 0099, from Dako, dilution 1∶1500), DEFA5 (mouse monoclonal anti-human alpha defensin NP5 Ab, clone nr 8C8, code ab62757, from Abcam plc, Cambridge, UK, dilution 1∶250) and DEFB1 (rabbit polyclonal anti-human beta defensin 1, kindly provided by Dr. Tomas Ganz, UCLA, Los Angeles, USA, dilution 1∶200). After two times 5 min of washing with PBS, the sections were incubated for 30 min with the secondary antibody, PowerVision Poly-HRP-anti-mouse/rabbit/rat IgG (Klinipath BVBA, Olen, Belgium), followed by 2 times 5 min washing with PBS. The complex was stained with 3-amino-9-ethylcarbazole for 10 min. After color development, the sections were rinsed in distilled water and were counterstained with Mayer's haematoxylin for 1 min. After washing with distilled water, the sections were mounted in glycerol medium (BDH, Dorset, UK). As negative controls, cryostat sections were processed as described, however without addition of the primary antibody. The stains were evaluated by two pathologists (KG and GDH) and the location of the staining product in the tissues was noted.

## Results

### AMP expression in intestinal mucosa from IBD patients before and after first infliximab treatment

**Figure 1:**
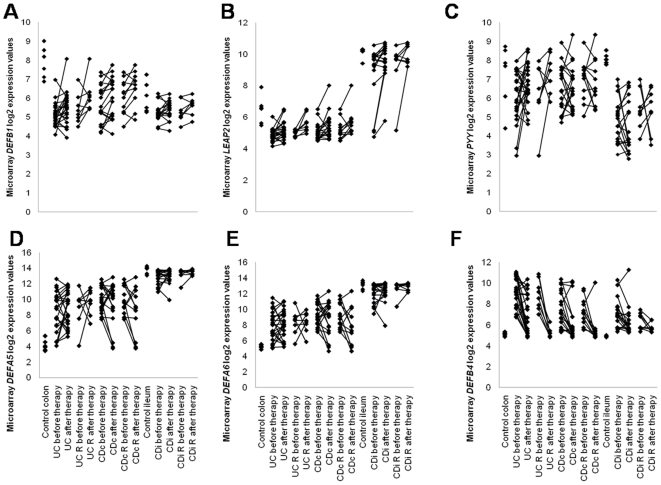
Individual microarray log2 expression values for selected AMP genes in intestinal mucosa of IBD patients before and after infliximab treatment and controls. (**A**) *DEFB1* (probe set 210397_at), (**B**) *LEAP2* (probe set 1552362_a_at), (**C**) *PYY* (probe set 207080_s_at), (**D**) *DEFA5* (probe set 207529_at), (**E**) *DEFA6* (probe set 207814_at) and (**F**) *DEFB4* (probe set 207356_at). Lines between 2 points represent the change in expression before and after treatment for one patient. R: responders.

Pairwise comparisons were performed for intestinal mucosal mRNA expression of 81 AMP genes between controls and patients before and after infliximab treatment in UC, CDc, IBDc (UC and CDc) and CDi, using LIMMA. The results (FC, p-value and FDR) of the performed comparative analyses for all the probe sets encoding AMP genes are given in Table S2. Figure 1A–F shows the individual microarray expression values for *DEFB1*, *LEAP2*, *PYY*, *DEFA5*, *DEFA6 and DEFB4*, respectively.

#### Colonic expression

First, we investigated whether there were differences in gene expression of AMPs in UC compared with CDc. Although no differences in expression of AMP genes at baseline reached significance comparing UC and CDc, we noted, however, that *DEFB4* mRNA expression was non-significantly increased in UC at baseline as compared with CDc at baseline (FC = 3.08, FDR = 0.18) (Table S2).

Next, we studied the AMP gene expression in inflamed colonic mucosa of IBD patients and the effect of infliximab treatment on the AMP gene expression. Table 2 shows the FC of the probe sets encoding AMP genes that were significant in one of the performed comparative analyses in UC, CDc and IBDc.

**Table 2:** Fold change of the probe sets encoding AMP genes that were significant (>2-fold change and FDR<0.05, underlined) in one of the performed comparative analyses in UC, CDc and IBDc.

		before T vs control colons			R after T vs control colons			NR after T vs control colons			R after T vs R before T		
Probe Set ID	Gene Symbol	UC	CDc	IBDc	UC	CDc	IBDc	UC	CDc	IBDc	UC	CDc	IBDc
205033_s_at	*DEFA1–A3*	1.60*	1.51	1.56*	0.97	0.96	0.96	1.34	2.39*	1.60	0.76	0.78	0.77*
207529_at	*DEFA5*	19.17*	64.58*	32.79*	47.12*	19.18	28.00*	18.45*	134.90*	33.81*	1.60	0.35	0.66
207814_at	*DEFA6*	6.42*	16.91*	9.85*	8.71	6.34	7.25	6.16*	29.53*	9.92*	1.03	0.41	0.60
210397_at	*DEFB1*	0.15*	0.21*	0.17*	0.29	0.39	0.35*	0.15*	0.16*	0.16*	1.61	1.34	1.45*
207356_at	*DEFB4*	17.29*	5.61*	10.52*	1.12	1.37	1.26	6.76*	4.28	5.88*	0.08*	0.34	0.19*
1555745_a_at	*LYZ*	17.57*	18.12*	17.81*	4.57	2.70	3.37	12.73*	28.02*	16.19*	0.24*	0.26*	0.25*
213975_s_at	*LYZ*	7.27*	7.45*	7.35*	3.59	2.97	3.22*	7.19*	8.17*	7.47*	0.48*	0.50*	0.49*
1552362_a_at	*LEAP2*	0.34*	0.38*	0.36*	0.55	0.56	0.56	0.32*	0.38*	0.34*	1.54*	1.39	1.45*
41469_at	*PI3*	11.73*	7.74*	9.76*	2.98	2.47	2.67	7.75*	7.55*	7.69*	0.23*	0.33*	0.29*
203691_at	*PI3*	11.03*	7.09*	9.08*	2.57	2.13	2.30	7.05*	6.89*	7.00*	0.19*	0.31*	0.26*
204971_at	*CSTA*	5.73*	5.29*	5.53*	1.31	1.78	1.57	3.64*	7.17*	4.48*	0.39*	0.54	0.47*
205916_at	*S100A7*	3.93*	2.29	3.09*	1.03	1.38	1.22	3.07	1.35	2.39	0.35*	0.63	0.49*
214370_at	*S100A8*	3.54*	2.37*	2.96*	0.99	1.11	1.06	2.64*	4.73*	3.15*	0.47*	0.77	0.63*
202917_s_at	*S100A8*	119.97*	65.89*	92.06*	1.65	2.26	1.98	39.87*	206.56*	65.78*	0.03*	0.08*	0.05*
203535_at	*S100A9*	8.24*	4.84*	6.51*	0.83	1.11	0.98	4.79*	12.20*	6.36*	0.20*	0.45*	0.32*
205863_at	*S100A12*	5.17*	3.35	4.27*	0.92	0.98	0.96	2.85	9.27*	4.08*	0.42	0.73	0.58*
202912_at	*ADM*	4.19*	2.95*	3.59*	1.34	1.60	1.48	2.95*	3.33*	3.06*	0.34*	0.56	0.45*
206390_x_at	*PF4*	4.04*	2.50*	3.26*	1.43	1.63	1.54	3.09*	2.52*	2.90*	0.33*	0.69	0.51*
214146_s_at	*PPBP*	3.08*	1.96	2.52*	1.01	1.25	1.14	2.65	3.86*	2.97*	0.44*	0.92	0.67
212067_s_at	*C1R*	4.72*	4.66*	4.69*	1.51	1.13	1.28	3.58*	6.56*	4.30*	0.51*	0.37*	0.43*
218232_at	*C1QA*	3.23*	3.21*	3.22*	1.30	0.95	1.08	2.78*	3.92*	3.09*	0.49	0.34*	0.40*
203649_s_at	*PLA2G2A*	4.64*	5.24*	4.89*	3.13	3.91	3.56*	4.51*	4.72*	4.57*	0.61	0.73	0.67*
202018_s_at	*LTF*	4.50*	3.02*	3.77*	1.20	1.63	1.43	3.07	2.41	2.85	0.41*	0.68	0.55*
212531_at	*LCN2*	17.66*	13.34*	15.60*	4.31	5.09	4.75	11.03*	13.18*	11.64*	0.21*	0.38	0.30*
205815_at	*REG3A*	43.82*	60.29*	50.45*	8.12	12.13	10.25	22.93*	114.88*	37.45*	0.16	0.25	0.20*
210037_s_at	*NOS2A*	7.86*	5.22*	6.56*	1.19	1.63	1.43	5.03*	4.07*	4.72*	0.15*	0.28*	0.22*
220104_at	*ZC3HAV1*	2.39*	2.01*	2.22*	1.12	1.53	1.34	2.19*	2.63*	2.31*	0.53*	0.75	0.65*

*: FDR<0.05, underline: significant (>2-fold change and FDR<0.05), R: responders; NR: non-responders, T: treatment.

*DEFB1* and *LEAP2* were the only AMP genes that showed a more than 2-fold and significantly reduction in mRNA expression in inflamed colon for both UC and CDc patients before infliximab treatment as compared to control colons. Moreover, *DEFB1* mRNA expression remained significantly more than 2-fold lower in IBDc responders after infliximab treatment compared to control colons (Table 2). A number of AMP genes (*PYY*, *NTS*, *NPY*, *KNG1*, *CST3*, *HIST1H2AB/HIST1H2AE* and *GRN*) showed a more than 1.5-fold decreased expression in active IBDc before infliximab treatment as compared with control colons, but they were not significant using our predefined significance criteria (Table S2). The expression of *LEAP2*, *NTS*, *and HIST1H2AB/HIST1H2AE* remained more than 1.5-fold non-significantly lower after therapy in IBDc responders compared to control colons (Table S2).

In contrast, the mRNA expression of 21 AMP genes (*DEFA5*, *DEFA6*, *DEFB4*, *LYZ*, *PI3*, *S100A7*, *S100A8*, *S100A9*, *S100A12*, *PF4*, *PPBP*, *CSTA*, *ADM*, *C1R*, *C1QA*, *PLA2G2A*, *LTF*, *LCN2*, *REG3A*, *NOS2A* and *ZC3HAV1*) was more than 2-fold significantly increased before treatment in active IBDc as compared with control colons. Most of the AMP genes upregulated at baseline in active IBDc decreased significantly more than 2-fold after infliximab treatment in IBDc responders when compared to their baseline samples. However, expression levels of *DEFA5*, *LYZ* and *PLA2G2A* mRNA remained more than 2-fold significantly higher in the IBDc responders after treatment compared to control colons (Table 2).

In non-responders to infliximab, no significant changes in AMP genes were found after infliximab treatment as compared with their baseline samples in UC, CDc and IBDc (Table S2). As compared with control colons, the AMP genes that were dysregulated at baseline remained dysregulated after treatment in non-responders (Table 2).

#### Ileal expression

Table 3 shows the FC of the probe sets encoding AMP genes that were significant in one of the performed comparative analyses with CDi.

**Table 3:** Fold change of the probe sets encoding AMP genes that were significant (>2-fold change and FDR<0.05, underlined) in one of the performed comparative analyses with CDi.

Probe Set ID	Gene Symbol	CDi before T vs CDc before T	CDi before T vs control ileums	CDi R after T vs control ileums	CDi NR after T vs control ileums
207529_at	*DEFA5*	6.60*	0.53	0.84	0.45
207814_at	*DEFA6*	7.18*	0.53	0.86	0.42
207356_at	*DEFB4*	0.71	4.61*	1.81	3.46
1552362_a_at	*LEAP2*	13.38*	0.42	0.96	0.61
203021_at	*SLPI*	0.18*	4.06*	2.22	3.46
41469_at	*PI3*	0.12*	5.04*	1.72	2.98
203691_at	*PI3*	0.11*	4.44*	1.17	2.47
205916_at	*S100A7*	0.45*	0.96	0.86	0.96
202917_s_at	*S100A8*	1.10	36.95*	2.41	34.58*
203535_at	*S100A9*	0.92	5.36*	1.38	4.80
206291_at	*NTS*	33.05*	0.13	0.40	0.13*
204260_at	*CHGB*	1.08	0.44*	0.69	0.47
211253_x_at	*PYY*	0.68*	0.37*	0.43*	0.35*
207080_s_at	*PYY*	0.29*	0.10*	0.15*	0.05*
202912_at	*ADM*	0.36*	3.30*	1.53	2.42
206390_x_at	*PF4*	0.60*	1.82*	1.46	2.15*
205500_at	*C5*	2.14*	1.73	1.66	1.66
204018_x_at	*HBA1/HBA2*	2.74*	1.42	1.25	3.16
211699_x_at	*HBA1/HBA2*	2.83*	1.42	1.26	3.18
217414_x_at	*HBA1/HBA2*	2.76*	1.41	1.27	3.33
209458_x_at	*HBA1/HBA2*	2.82*	1.37	1.22	3.07
214414_x_at	*HBA1/HBA2*	2.94*	1.36	1.35	3.23
211745_x_at	*HBA1/HBA2*	2.87*	1.40	1.25	3.18
212531_at	*LCN2*	0.63*	22.20*	10.54*	16.22*
205815_at	*REG3A*	7.85*	0.85	1.00	0.69
231661_at	*REG3G*	2.34*	0.55	1.07	0.46
210037_s_at	*NOS2A*	0.85	3.38*	3.02	2.74

*: FDR<0.05, underline: significant (>2-fold change and FDR<0.05), R: responders; NR: non-responders, T: treatment.

First, we studied the differential expression between CDi and CDc at baseline. At baseline, the mRNA expression levels of *DEFA5*, *DEFA6*, *LEAP2*, *NTS*, *C5*, *HBA1/HBA2*, *REG3A* and *REG3G* were more than 2-fold significantly upregulated in CDi as compared with CDc. The expression levels of *SLPI*, *PI3*, *S100A7*, *PYY* and *ADM* were more than 2-fold significantly downregulated at baseline in CDi as compared with CDc (Table 3).

Next, we investigated the effect of infliximab on AMP gene expression in inflamed ileal mucosa of CDi patients.

Only two AMP genes (*PYY* and *CHGB*) both belonging to the neuropeptide group showed a more than 2-fold significantly decreased expression in active CDi at baseline when compared to control ileums, and *PYY* expression remained more than 2-fold significantly lower in CDi responders after infliximab treatment compared to control ileums (Table 3). Other AMP genes that showed a more than 1.5-fold non-significantly decreased expression in active CDi at baseline as compared with control ileums were *DEFA5*, *DEFA6*, *DEFB1*, *LEAP2*, *NPY*, *NTS*, *CHGA*, *KNG1*, *REG3G* and *HMGB1* (Table S2). The expression of the latter genes and also *CHGB* remained more than 1.5-fold non-significantly lower after therapy in CDi responders than in control ileums, except for *DEFA5*, *DEFA6* and *LEAP2* (Table S2).

Eight AMP genes (*DEFB4*, *SLPI*, *PI3*, *S100A8*, *S100A9*, *ADM*, *LCN2* and *NOS2A*) were more than 2-fold significantly upregulated in CDi at baseline in comparison with control ileums. Only *LCN2* mRNA expression remained more than 2-fold significantly increased after infliximab treatment in CDi responders as compared to control ileums (Table 3).

### The relationship between AMPs and epithelial integrity, inflammatory activity and Paneth cell mass

**Figure 2:**
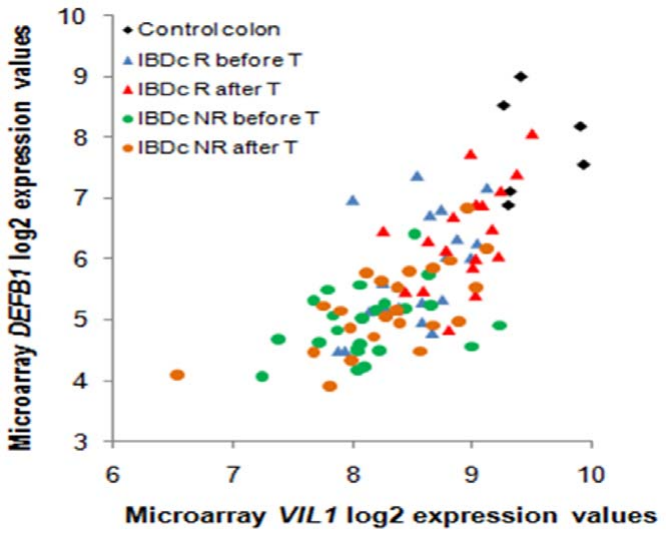
Scatterplot representing correlation between colonic microarray log2 expression values of *DEFB1* and *VIL1*. R: responders; T: treatment.

We also investigated the relationship between the mRNA expression of AMPs that remained downregulated (*DEFB1* in IBDc and *PYY* in CDi) or upregulated (*DEFA5*, *LYZ* and *PLA2G2A* in IBDc, and *LCN2* in CDi) after therapy in responders to infliximab and markers for epithelial integrity and for inflammatory activity, respectively. The correlations were analyzed with the Spearman's Rank Correlation test using the microarray log2 mRNA expression values, and a p-value<0.05 was considered significant (Table 4). The ileum and colon samples were analysed separately. The mRNA levels of the probe set 205506_at representing *VIL1*, a marker of epithelial cell content, correlated significantly with the mRNA levels of *DEFB1* in the colon (Figure 2), and the correlation with *PYY* was borderline significant (p-value = 0.06) in the ileum. The mRNA levels of the probe set 202859_x_at representing *IL8*, an inflammatory marker, were used to evaluate the correlation of the mRNA levels of the upregulated AMPs with the inflammatory activity. For the upregulated AMPs, the colonic mRNA levels of *DEFA5*, *LYZ* and *PLA2G2A* showed a positive significant correlation with the colonic mRNA levels of *IL8*, and there was a positive significant correlation between the ileal mRNA levels of *LCN2* and *IL8*.

**Table 4:** Results (correlation coëfficient and p-value) of the Spearman's rank correlation analyses between the remaining dysregulated AMPs after treatment in responders, *VIL1* and *IL8*, and between alpha-defensins and *PLA2G2A*, using the microarray log2 normalized expression values of the probe sets representing these genes.

	Ileum samples (n = 42)		Colon samples (n = 91)	
Spearman's rank correlation analyses	correlation coëfficient	p-value	correlation coëfficient	p-value
***DEFB1*** ** (210397_at) and ** ***VIL1*** ** (205506_at)**	0.332	0.032	0.708	<0.001
***PYY*** ** (207080_s_at) and ** ***VIL1*** ** (205506_at)**	0.289	0.064	0.409	<0.001
***DEFA5*** ** (207529_at) and ** ***IL8*** ** (202859_x_at)**	−0.416	0.006	0.22	0.036
***PLA2G2A*** ** (203649_s_at) and ** ***IL8*** ** (202859_x_at)**	0.175	0.267	0.335	0.001
***LYZ*** ** (213975_s_at) and ** ***IL8*** ** (202859_x_at)**	0.226	0.151	0.746	<0.001
***LCN2*** ** (212531_at) and ** ***IL8*** ** (211506_s_at)**	0.477	0.001	0.477	<0.001
***DEFA5*** ** (207529_at) and ** ***PLA2G2A*** ** (203649_s_at)**	0.466	0.002	0.28	0.007
***DEFA6*** ** (207814_at) and ** ***PLA2G2A*** ** (203649_s_at)**	0.565	<0.001	0.31	0.003
***DEFA5*** ** (207529_at) and ** ***VIL1*** ** (205506_at)**	0.503	0.001	−0.098	0.353
***DEFA6*** ** (207814_at) and ** ***VIL1*** ** (205506_at)**	0.43	0.004	−0.125	0.237

For *DEFA5* and *DEFA6*, we observed a non-significantly decreased expression in active CDi. In the ileum, *PLA2G2A* can be used as a marker of Paneth cell mass. Therefore, we studied the correlation of the microarray log2 expression levels in the ileum between the alpha-defensins (*DEFA5* and *DEFA6*) and *PLA2G2A*. A positive significant relationship was identified between the mRNA levels of the alpha-defensins and *PLA2G2A* in the ileum. We found also a positive correlation between the mRNA levels of the alpha-defensins and *VIL1* in the ileum.

### Validation of the microarray data by qPCR

The differential mRNA expression of *DEFB1*, *LEAP2*, *PYY*, *DEFA5*, *DEFA6 and DEFB4*, observed by microarray analysis, was confirmed by qPCR (Figure S1, Table S3).

Moreover, using qPCR we found more genes that were significantly different between the groups. However, it must be noted that the statistical criteria used for defining significance by qPCR (P-value_Mann-Whitney test/Wilcoxon signed-rank test_<0.05) were less strict than the criteria used for microarray data significance by LIMMA (>2-fold change and FDR<0.05). Also, fewer samples were studied by qPCR than by microarray analysis.

### Protein localization by immunohistochemistry

Immunohistochemistry was performed to determine where DEFB1, DEFA5 and LYZ are expressed in the colonic and ileal mucosa of healthy controls and IBD patients before and after infliximab therapy.

**Figure 3:**
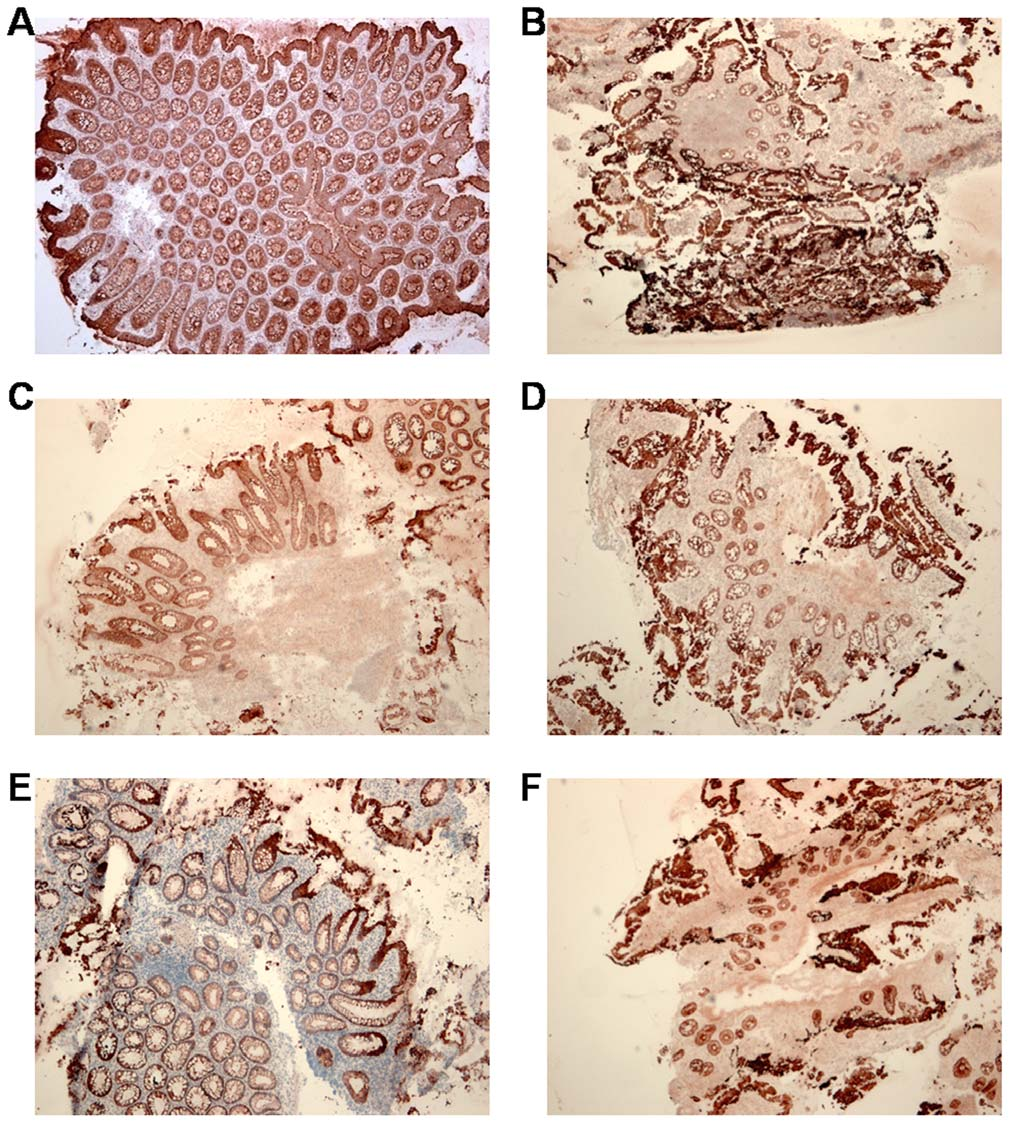
Immunohistochemical detection of DEFB1 protein in intestinal mucosa. Intense immunostaining of epithelial cells at the mucosal surface in normal colon (**A**) and ileum (**B**). Severely reduced DEFB1 expression in UC (**C**) and CDi (**D**) before infliximab treatment. Partial restoration of epithelial cell mass with DEFB1 staining in UC (**E**) and CDi (**F**) in responders to infliximab therapy (original magnification (OM): ×50).

In the normal intestine, DEFB1 was expressed in the epithelial cell compartment, with accentuation at the surface of the biopsies (Figure 3A–B). A similar expression pattern was seen in biopsies from IBD patients, both before and after infliximab treatment. We observed important epithelial cell loss in UC, CDc and CDi biopsies before treatment, leading to an overall diminished DEFB1 expression in these samples (Figure 3C–D). This loss of protein expression was only partly restored in responders after infliximab therapy (Figure 3E–F).

**Figure 4:**
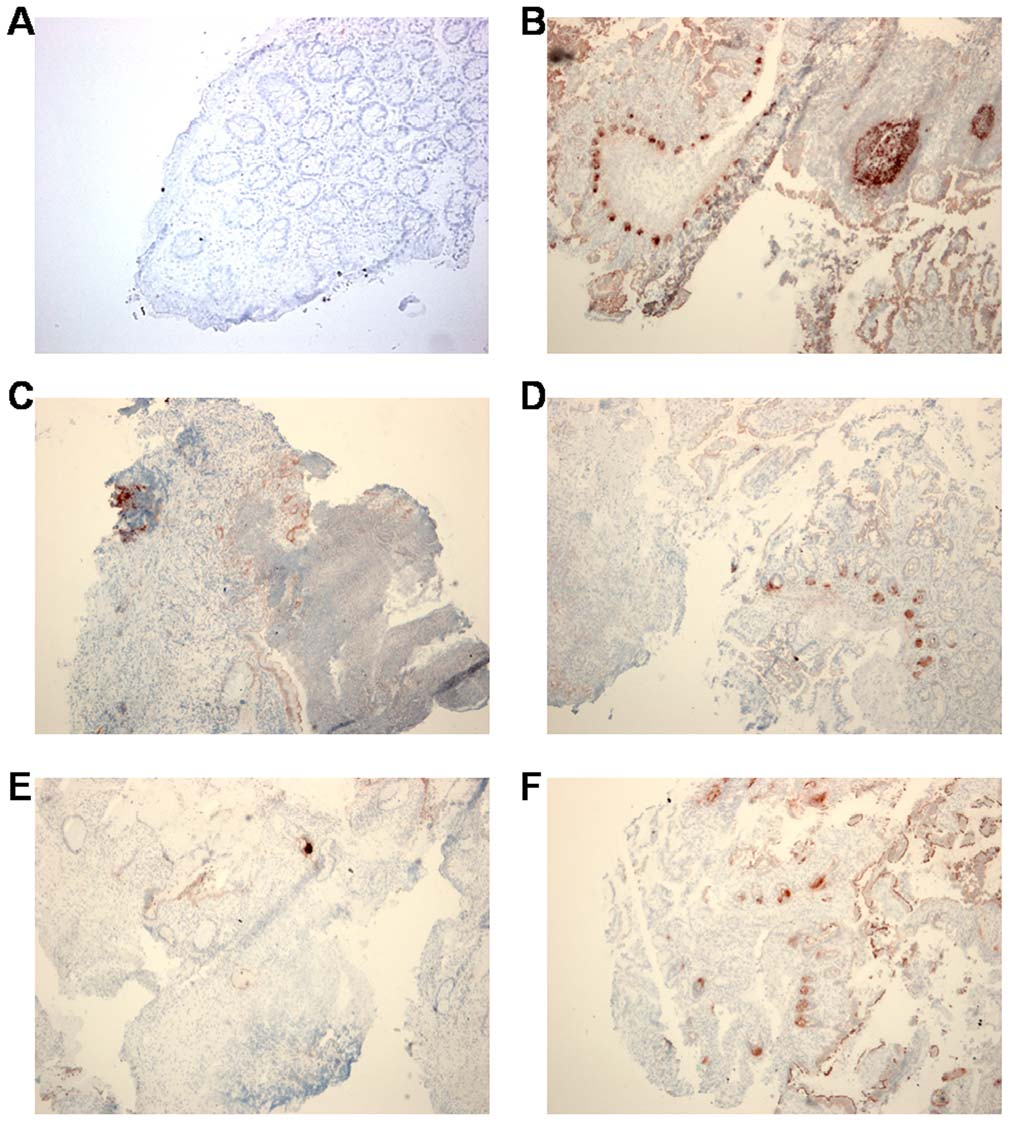
Immunohistochemical detection of DEFA5 protein in intestinal mucosa. No immunostaining can be seen in normal colon (**A**), while Paneth cells and follicle centers in Peyer's patches are immunoreactive in normal ileum (**B**). Mucosal defects with inflammatory cells staining for DEFA5 in untreated UC (**C**) and Paneth cell loss with diminished DEFA5 staining in active CDi (**D**). Rare DEFA5-positive metaplastic Paneth cells in colonic mucosa of UC responders after infliximab (**E**). No difference in DEFA5 immunoreactivity when comparing CDi responders with untreated CDi (**F**). (OM (**A**): ×100; OM (**B–F**): ×50).

DEFA5 was not expressed in the normal colonic mucosa, whereas in the ileum it was mainly seen in the Paneth cells (Figure 4A–B). Colonic biopsies from untreated IBD patients frequently showed extensive epithelial defects and granulation tissue with inflammatory cells, which stained positive for DEFA5 (Figure 4C). Epithelial cell loss was also observed in the ileum of untreated CD patients. The overall diminished DEFA5 expression in these biopsies was related to a decreased number of crypts (Figure 4D). After successful infliximab therapy, there was some regeneration of the epithelial cell compartment both in the colon and ileum. DEFA5 expression in the IBD colon shifted partly from inflammatory cells to rare metaplastic Paneth cells (Figure 4E). There were no obvious differences in expression pattern of DEFA5 in ileal CD after therapy when compared with untreated CDi patients (Figure 4D and F).

In healthy controls, LYZ was mainly expressed in Paneth cells in the ileum and lamina propria mononuclear cells both in the ileum and colon. Intestinal mucosal inflammation in untreated IBD patients was characterized by an influx of numerous LYZ-expressing polymorphonuclear leukocytes and histiocytes. After successful infliximab therapy, the density of mucosal inflammatory cells decreased, which resulted in a slightly diminished LYZ expression (data not shown).

## Discussion

The most commonly accepted hypothesis on the IBD etiology is that abnormalities in the innate immune response by the mucosa cause a loss of tolerance to commensal microbiota and alterations in the composition of the gut microbiota. As a consequence for this defect, the host immune system is overwhelmed by bacterial antigens which lead to chronic immune-mediated intestinal injury. There is evidence that expression and regulation of defensins, a class of antimicrobial peptides (AMPs), produced in the intestinal mucosa are altered in IBD [12]. Whether the abnormalities in defensins in the mucosa in IBD are primary defects or are the consequence of inflammation is still debated. Moreover, other AMPs may also play a role in the pathogenesis of chronic inflammation. A large number of AMPs have been identified, including the “classic” AMPs (e.g. defensins) and other molecules that were first discovered for other biological activities (e.g. neuropeptides) [34]. The main goal of the present study was to investigate the influence of downregulation of inflammation by infliximab, the IgG1 monoclonal antibody to TNF-alpha, on the expression of AMPs in ileal and colonic CD and in UC in comparison with normal controls, using microarray technology.

In this study we found no significant differences at baseline in AMP expression in Crohn's colitis mucosa in comparison with UC mucosa using our strict predefined significance criteria. Our microarray studies further showed that 21 AMPs were upregulated in inflamed colon from IBD patients before infliximab treatment in comparison with normal colons. For most of these upregulated AMPs, colonic expression almost completely normalized after treatment in IBD responders compared to their baseline samples. Only the colonic expression of *DEFA5*, *LYZ* and *PLA2G2A* remained significantly higher after treatment in IBD responders compared to control colons. Increased expression of the former AMPs is likely due to Paneth cell metaplasia which is readily found in active colitis [12]. Noble et al. [16] also showed an increased *DEFA5* and *DEFA6* expression in UC colon, and linked this to Paneth cell metaplasia. Next, two AMPs were significantly downregulated in active IBD colitis in comparison with normal colons, namely *LEAP2* and *DEFB1*. Expression of *LEAP2* [35] in IBD has not formerly been described. The *LEAP2* colonic expression increased non-significantly in IBD responders after infliximab therapy but did not normalize completely. *DEFB1* colonic expression remained significantly decreased in IBD responders after infliximab therapy. The expression of a number of AMP genes were dysregulated at baseline between ileal and colonic CD. In active ileal Crohn's disease before treatment 8 AMPs were upregulated versus control ileums, whereas *DEFA5*, *DEFA6*, *DEFB1* and *LEAP2* were downregulated although not significantly. Only *PYY* and *CHGB* were significantly downregulated in ileal CD prior to therapy as compared to control ileums, and *PYY* remained significantly lower after therapy in ileal CD responders. Quantitative real-time reverse-transcription PCR and immunohistochemistry confirmed the microarray data.

In this study the close relationship between the remaining downregulated AMPs after treatment (*DEFB1* and *PYY*) with villin 1, a marker of epithelial cell mass, strongly suggests that the decreased expression of these AMPs is the consequence of epithelial cell and enterochromaffine cell damage and loss. It was also shown with immunohistochemistry that although there is epithelial layer restoration after treatment with infliximab in responders, this restoration is incomplete and epithelial cell mass is still decreased in comparison with normals. Similarly, in the ileum the expression of alpha-defensins (*DEFA5* and *DEFA6*) correlated well with the expression of *PLA2G2A*, a marker for Paneth cells, which suggests that Paneth cell loss is responsible for the non-significantly decreased alpha-defensin expression in ileal CD. This is in accordance with the data published by Simms et al. [15].

We also found no differences in expression of AMPs between patients with *CARD15* mutation(s) and patients without *CARD15* mutation (data not shown) similar to the findings by Simms et al. [15]. These authors found no significant relationship between the alpha-defensin (*DEFA5* and *DEFA6*) expression and *CARD15* mutation in ileal Crohn's disease. Because of the low number of observations in our present study, we think further studies are necessary.

Our study suggests that decreased expression of antimicrobial peptides in IBD is not a primary defect causing the disease but a consequence of epithelial cell loss in the ileum and colon, and loss of Paneth cells in the ileum in an active phase of the disease. We further hypothesize that decreased secretion of AMPs as a consequence of epithelial damage in established IBD may contribute to the perpetuation of inflammation because ongoing bacterial invasion of the mucosa cannot be controlled, even despite Paneth cell metaplasia resulting in an increased production of some of the AMPs in the colon.

## Supporting Information

Appendix S1 A glossary of terms used in the methods.
(0.03 MB DOC)

Figure S1 qPCR analysis of DEFB1 (A), LEAP2 (B), PYY (C), DEFA5 (D), DEFA6 (E) and DEFB4 (F) in intestinal mucosa of IBD patients before and after first infliximab treatment. A line between 2 points represents the change in expression before and after treatment for one patient. R: responders.
(2.18 MB TIF)

Table S1 A list of the 81 AMP proteins that were investigated in the present study.
(0.03 MB XLS)

Table S2 The microarray analysis results (FC, p-value and FDR) for all the probe sets representing AMP genes of the performed comparative analyses by LIMMA in and between UC, CDc, IBDc and CDi. R: responders, NR: non-responders.
(0.23 MB XLS)

Table S3 The results (p-value) from the comparative analyses of the qPCR data of DEFB1, LEAP2, PYY, DEFA5, DEFA6 and DEFB4 in intestinal mucosa of IBD patients before and after first infliximab treatment. R: responders, NR: non-responders.
(0.02 MB XLS)
