# Supplementary material for: Mucosal Gene Expression of Antimicrobial Peptides in Inflammatory Bowel Disease Before and After First Infliximab Treatment
Source: PLoS One. 2009 Nov 24;4(11):e7984. doi: 10.1371/journal.pone.0007984 (PMC2776509; doi:10.1371/journal.pone.0007984)
Supplement: Appendix S1 — A glossary of terms used in the methods. (0.03 MB DOC) [file pone.0007984.s001.doc]

**GLOSSARY**

**.cel file:** The extension of the cell intensity file. It is created from the data file and it contains a single intensity value for each probe cell delineated by a grid.

**Bioconductor:** A free, open source and open development software project for the analysis and comprehension of genomic data. It is based on the [statistical](http://en.wikipedia.org/wiki/Statistics) [R programming language](http://en.wikipedia.org/wiki/R_(programming_language)).

**False discovery rate (FDR):** An alternative measure for multiple testing error, introduced by Benjamini and Hochberg (1995). FDR is the expected proportion of false positives among the list of probe sets/genes declared to be differentially expressed.

**Gene expression profiling:** Measurement of the expression level of thousands of genes simultaneously in one RNA sample by using, for example, a microarray, a glass slide or membrane that is spotted or arrayed with DNA fragment or oligonucleotides that represent specific gene coding regions.

**Linear models for microarray data (LIMMA):** A bioconductor package for the analysis of gene expression microarray data, especially the use of linear models for analysing designed experiments and the assessment of differential expression. For our comparative analyses, we used Emperical Bayes moderated *t*-statistics in LIMMA which is more powerful for small samples sizes than standard *t*-tests.

**Mann-Whitney *U*-test:** A non-parametric pairwise comparison test for independent samples.

**Probe set:** A group of oligonucleotide-probe pairs designed to detect the expression level of one gene transcript. Two oligonucleotides (25 bases) designed to be complementary to a reference sequence, of which one has a mismatch at the 13th position, define a probe pair. Mismatch probes serve as control for cross-hybridization. A probe set on the Affymetrix Human Genome U133 Plus 2.0 Array contains 11 probe pairs.

**Robust multichip average (RMA) method:** A data pre-processing method to compute expression summery values for each probe set. The RMA method consists of three steps: background correction, quantile normalization and computing log2 intensity expression summary values for each probe set.

**Spearman’s Rank Correlation test:** A statistical technique to test the direction and strength of the relationship between two variables.

**Wilcoxon signed-rank test:** A non-parametric pairwise comparison test for paired samples.

**Quantitative real-time reverse-transcription polymerase chain reaction (qPCR):** A method to quantify mRNA in a sample.
