# Supplementary figures and images for: Mucosal Gene Expression of Antimicrobial Peptides in Inflammatory Bowel Disease Before and After First Infliximab Treatment
Source: PLoS One. 2009 Nov 24;4(11):e7984. doi: 10.1371/journal.pone.0007984 (PMC2776509; doi:10.1371/journal.pone.0007984)

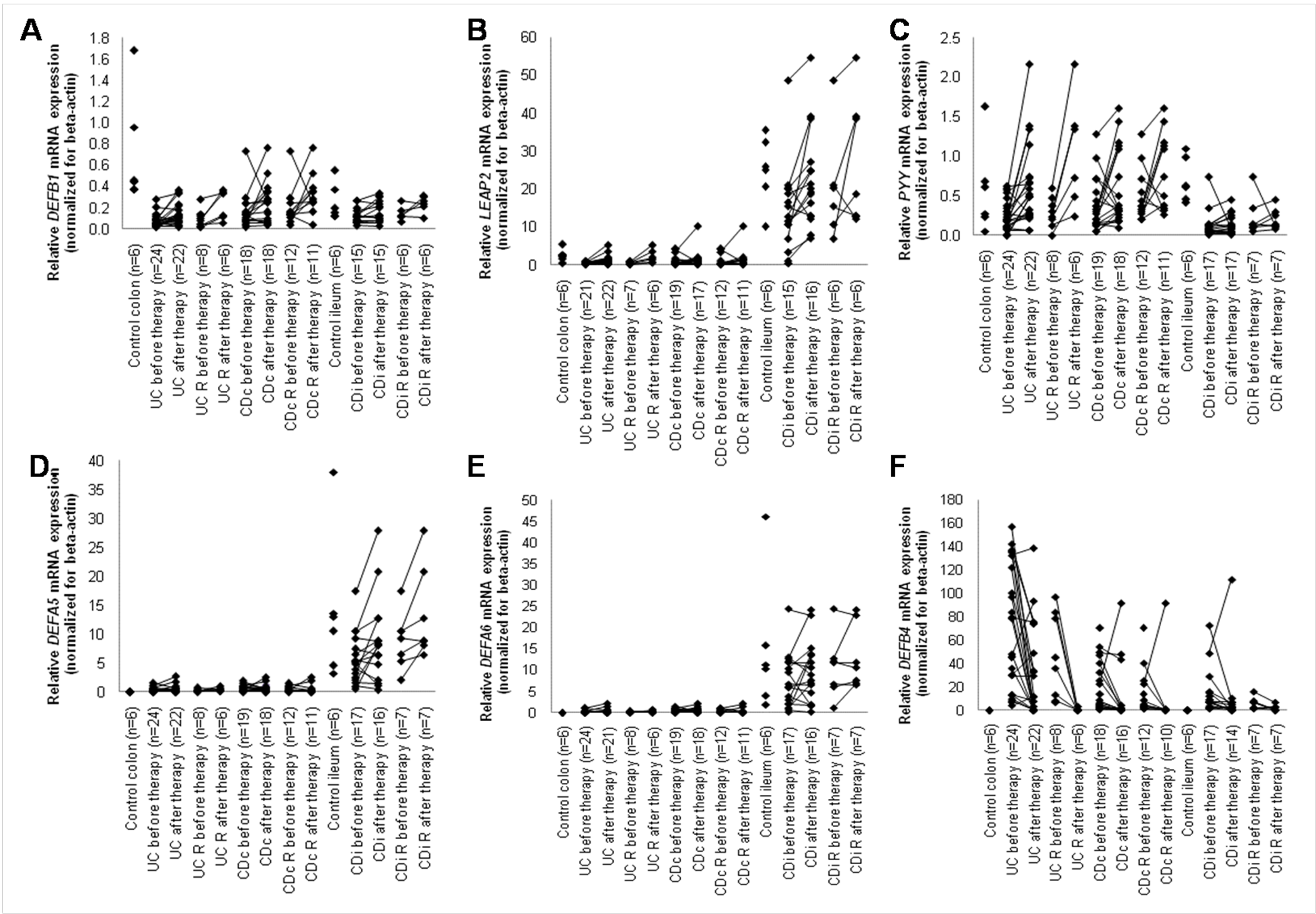

Supplement: Figure S1 — qPCR analysis of DEFB1 (A), LEAP2 (B), PYY (C), DEFA5 (D), DEFA6 (E) and DEFB4 (F) in intestinal mucosa of IBD patients before and after first infliximab treatment. A line between 2 points represents the change in expression before and after treatment for one patient. R: responders. (2.18 MB TIF) [file pone.0007984.s002.tif]
